# Designing for Engagement in Primary Health Education Through Digital Game-Based Learning: Cross-National Behavioral Evidence from the iLearn4Health Platform

**DOI:** 10.3390/bs15070847

**Published:** 2025-06-24

**Authors:** Evgenia Gkintoni, Emmanuella Magriplis, Fedra Vantaraki, Charitini-Maria Skoulidi, Panagiotis Anastassopoulos, Alexandra Cornea, Begoña Inchaurraga, Jaione Santurtun, Ainhoa de la Cruz Mancha, George Giorgakis, Kleri Kouppas, Stella Timotheou, Maria Jose Moreno Juan, Miren Muñagorri, Marta Harasiuk, Alfredo Garmendia Lopez, Efi Skoulidi, Apostolos Vantarakis

**Affiliations:** 1 Lab of Public Health, Department of Medicine, University of Patras, 26504 Patra, Greece; fedra2003@gmail.com (F.V.); avanta@upatras.gr (A.V.); 2 Lab of Dietetics and Quality of Life, Department of Food Science and Human Nutrition, Agricultural University of Athens, 11855 Athens, Greece; emagriplis@aua.gr; 3 p-Consulting, 26442 Patras, Greece; cms@p-consulting.gr (C.-M.S.); pga@p-consulting.gr (P.A.); ag@p-consulting.gr (A.G.L.); es@p-consulting.gr (E.S.); 4 FSLI, 030167 Bucharest, Romania; alexandra_cornea@yahoo.com; 5 Centro San Viator, 48190 Sopuerta, Spain; b.inchaurraga@sanviator.com (B.I.); j.santurtun@sanviator.com (J.S.); ainhoa.delacruz@sanviator.com (A.d.l.C.M.); 6 C.F.C.D.C. Centre for Competence, Development Cyprus Limited, Nicosia 1065, Cyprus; george@eurosc.eu; 7 Dimotiko Scholeio Agias Napas-Antoni Tsokkou, Ayia Napa 5330, Cyprus; kkouppas@hotmail.com (K.K.); stellakalli1@gmail.com (S.T.); 8 Errotu Taldea S.L.P., 20160 Donostia-San Sebastian, Spain; mjmorenojuan@gmail.com (M.J.M.J.); mmunagorri@errotu.com (M.M.); 9 OIC POLAND Foundation, WSEI University, 20-209 Lublin, Poland; marta.harasiuk@oic.lublin.pl

**Keywords:** digital game-based learning (DGBL), design evaluation, primary health education, cross-national implementation, user engagement analytics, educational technology design, teacher assessment, platform usability, design validation, health education interface design

## Abstract

This study evaluates design effectiveness in Digital Game-Based Learning (DGBL) for primary health education through systematic teacher assessment of the iLearn4Health platform. Rather than measuring educational transformation, the research investigates how DGBL design principles influence user engagement patterns and platform usability as evaluated by education professionals. The study contributes to design optimization frameworks for primary school digital health education applications by examining the distinction between DGBL and superficial gamification approaches in creating engaging educational interfaces. The iLearn4Health platform underwent comprehensive design evaluation by 337 teachers across 24 schools in five European countries (Greece, Cyprus, Romania, Poland, and Spain). Teachers served as design evaluators rather than end-users, assessing platform engagement mechanisms through systematic interaction analysis. The study employed multiple statistical approaches—descriptive analysis, correlation analysis, ANOVA, regression modeling, and cluster analysis—to identify design engagement patterns and their predictors, tracking completion rates, progress trajectories, and interaction time as indicators of design effectiveness. Design evaluation revealed a distinctive bimodal engagement distribution, with 52.8% of teacher–evaluators showing limited platform exploration (progress ratio 0.0–0.2) and 35.3% demonstrating comprehensive design assessment (progress ratio 0.8–1.0). A strong positive correlation (r = 0.95, *p* < 0.001) between time spent and steps completed indicated that design elements successfully sustained evaluator engagement. Multiple regression analysis identified initial design experience as the strongest predictor of continued engagement (β = 0.479, *p* < 0.001), followed by country-specific implementation factors (Romania vs. Cyprus, β = 0.183, *p* = 0.001) and evaluator age (β = 0.108, *p* = 0.049). Cluster analysis revealed three distinct evaluator profiles: comprehensive design assessors (35.3%), early design explorers (52.8%), and selective feature evaluators (11.9%). Cross-national analysis showed significant variations in design engagement, with Romania demonstrating 53% higher average progress ratios than Cyprus (0.460 vs. 0.301, *p* < 0.01). Teacher evaluation validates effective design implementation in the iLearn4Health platform for creating engaging primary health education experiences. The platform successfully demonstrates DGBL design principles that integrate health concepts into age-appropriate interactive environments, distinct from gamification approaches that merely overlay game elements onto existing content. Identifying initial engagement as the strongest predictor of sustained interaction highlights the critical importance of onboarding design in determining user experience outcomes. While this study establishes design engagement effectiveness through educator assessment, actual educational transformation and student learning outcomes require future implementation studies with primary school populations. The design validation approach provides essential groundwork for subsequent educational effectiveness research while contributing evidence-based design principles for engagement optimization in digital health education contexts.

## 1. Introduction

In recent years, the educational and health sectors have increasingly incorporated game elements into their practices, albeit through distinct design approaches that merit clear differentiation for effective implementation. Gamification—the application of game-design elements to non-game contexts—differs fundamentally from Digital Game-Based Learning (DGBL), which employs complete games explicitly designed for educational purposes. This critical distinction establishes the framework for our design evaluation investigation (Camacho-Sánchez et al., 2022; Q. Zhang & Yu, 2022; de Carvalho & Coelho, 2022; Dahalan et al., 2024).

Gamification typically incorporates standard mechanics such as points, leaderboards, rewards, and levels into non-gaming environments to enhance user motivation and engagement (Mohanty & Christopher, 2023; Oke et al., 2024; Zhao et al., 2021). Since approximately 2010, gamification has gained significant traction across multiple domains, including education, marketing, and public health. By contrast, DGBL involves comprehensive game experiences specifically developed to achieve learning objectives, where educational content is intrinsically woven into gameplay mechanics (Vijay & Jayan, 2023; Castellano-Tejedor & Cencerrado, 2024; Speelman et al., 2023; Zolfaghari et al., 2025).

The education sector has embraced both approaches, with gamification elements enhancing traditional learning platforms and DGBL offering immersive educational experiences. Similarly, the health promotion field has adopted these strategies—gamification appears in behavior-tracking applications and incentive systems, while health-focused games deliver educational content through interactive narratives and simulations (M. Xu et al., 2023; Shaheen et al., 2023; Abou Hashish et al., 2024).

Despite growing interest in both fields, there remains a significant gap in research that systematically evaluates design effectiveness across these distinct approaches in educational and health contexts (Rycroft-Smith, 2022; Cong-Lem et al., 2025; Taneja-Johansson & Singal, 2025). The current literature often conflates or treats these distinct concepts in isolation without rigorous design assessment frameworks. This lack of design evaluation methodology hinders the development of effective implementation strategies that could inform evidence-based design practices in both fields (Samala et al., 2025; Caprara & Caprara, 2022; Keyes & Platt, 2024).

This study focuses specifically on design evaluation and engagement optimization rather than educational transformation assessment. While the ultimate goal of health education platforms is to improve children’s knowledge and behavior, this research addresses the prerequisite step of design validation through systematic educator evaluation. We examine design effectiveness for creating engaging interactions that could support future educational implementation, without claiming to demonstrate educational transformation or student outcome improvement.

Our research examines explicitly the design characteristics of the iLearn4Health platform, an online DGBL application designed to optimize engagement in health literacy development among primary school students. This platform features modules addressing Healthy Dietary Habits, Physical Activity, Stereotypes, Accident Prevention, Internet Safety, and Sexual Health Education. Unlike gamified applications that merely add game elements to existing content, iLearn4Health represents a comprehensive DGBL design environment where health concepts are integrated into the core gameplay mechanics (Halkiopoulos & Gkintoni, 2024).

This study aims to analyze how DGBL design principles—rather than gamification approaches broadly—contribute to user engagement patterns in health education contexts. By establishing clear theoretical boundaries between gamification and DGBL design approaches, we can more accurately assess how game-based interface design enhances sustained interaction and usability in health education platforms. Furthermore, we investigate how data-driven design insights can inform iterative optimization processes that align educational game interfaces with engagement objectives (Muniandy et al., 2023; All et al., 2021; Antonopoulou et al., 2022; K. Wang et al., 2023).

Rather than measuring educational effectiveness or student outcomes, this investigation employs teacher evaluation as a design validation methodology. Teachers serve as informed design evaluators who assess platform engagement mechanisms, interface usability, and age-appropriateness for the target demographic. This approach provides critical design feedback while acknowledging that educational transformation requires subsequent implementation studies with primary school populations.

Through this focused design evaluation approach, we contribute to a more nuanced understanding of how game-based design strategies—specifically gamification versus DGBL—can effectively support engagement objectives in health education contexts. This design-centered analysis addresses a critical need in the literature for systematic evaluation methodologies when assessing game-related interface design across these intersecting fields (Sáiz-Manzanares et al., 2021; Feldhacker et al., 2025; Vázquez-Calatayud et al., 2024; Gkintoni et al., 2024b).

The study makes three primary contributions to educational technology design research: First, it establishes a systematic approach for evaluating DGBL design effectiveness through educator assessment, providing a replicable framework for design optimization in educational contexts. Second, it identifies specific design factors that predict sustained user interaction, contributing evidence-based principles for interface optimization in primary school health education applications. Third, it examines how design effectiveness varies across different cultural and educational contexts, informing adaptive design strategies for international implementation.

This design evaluation study establishes engagement effectiveness but does not measure educational transformation or student learning outcomes. The findings provide essential groundwork for future educational effectiveness research while contributing to design optimization frameworks for digital health education. Subsequent implementation studies with primary school students are necessary to validate whether design engagement translates into educational impact and behavioral change. By maintaining clear boundaries between design validation and educational effectiveness assessment, this research offers valuable insights into engagement-focused design principles while appropriately positioning future research needs for comprehensive educational evaluation.

## 2. Literature Review

### 2.1. Theoretical Frameworks Distinguishing Gamification and DGBL in Education and Health Promotion

Theoretical underpinnings that distinguish gamification and Digital Game-Based Learning (DGBL) are essential for effective application in both educational and health settings (del Cura-González et al., 2022; Gkintoni et al., 2025b; Gupta & Goyal, 2022). Gamification heavily borrows from motivational theories and, more specifically, Self-Determination Theory (SDT), which postulates autonomy, competence, and relatedness as basic psychological needs. When game design mechanics like points, badges, and leaderboards are translated into non-game settings, they can potentially facilitate or undermine these inherent motivational needs (Gao, 2024; H. Oliveira et al., 2024; Nivedhitha et al., 2025; Y. Chen & Zhao, 2022).

On the other hand, DGBL has stronger roots in constructivist learning theory, in which learning is achieved through experience and problem-solving in whole game worlds (Tyack & Mekler, 2024; Breien & Wasson, 2021). Whereas gamification incorporates game mechanics into existing tasks, DGBL incorporates curricular material into the gameplay itself, with an opportunity to engage more deeply with learning content (Papakostas, 2024; R. Zhang et al., 2025; Nadeem et al., 2023).

In health promotion specifically, both the Theory of Planned Behavior (TPB) and Social Cognitive Models provide frameworks through which both approaches can influence health behaviors in different ways (Höyng, 2022; Yang et al., 2021; Luo, 2022). Gamification targets specific behavior through the use of extrinsic rewards, while DGBL can potentially create immersive storylines that embed health concepts into significant experiences (Kim & Castelli, 2021; Macey et al., 2025; Riar et al., 2022).

Research with primary school populations, the specific target demographic for the iLearn4Health platform, demonstrates particular developmental considerations that affect the efficacy of both approaches (Nordby et al., 2024; Xiong et al., 2022; Ghosh et al., 2022). Children aged 6–12 respond differently to game elements than adolescents or adults, with greater responsiveness to visual rewards, narrative engagement, and playful interactions. This developmental distinction is critical when designing interventions specifically for primary school students versus general populations, as the cognitive and motivational processes differ substantially across age groups (Hassan et al., 2021; Henry et al., 2021; Masi et al., 2021).

Experimental comparisons between minimally gamified applications and those with comprehensive game elements demonstrate that theoretical grounding significantly impacts outcomes (Leitão et al., 2022a; Dah et al., 2024; Kniestedt et al., 2022). These comparisons reveal that superficial implementation of game elements without consideration of age-appropriate motivational factors often fails to produce sustained engagement, particularly among primary school children who require more concrete and immediate feedback systems than adult users (Torresan & Hinterhuber, 2023; Leitão et al., 2022b; Tzachrista et al., 2023).

### 2.2. Design Principles for Effective Gamification vs. DGBL in Primary School Health Education

The design principles for gamification differ substantially from those for DGBL, particularly when targeting primary school children for health education. User-centered design takes on heightened importance with younger populations, as their cognitive abilities, attention spans, and motivational triggers vary significantly from adolescents or adults (Pitic & Irimiaș, 2023; Wiljén et al., 2022).

For gamification approaches with primary school children, effective design principles include the following: (1) immediate and concrete feedback mechanisms, (2) simple reward structures that avoid overwhelming cognitive load, (3) age-appropriate challenges that match developmental capabilities, and (4) social comparison elements calibrated to minimize adverse competitive effects. These principles address the specific needs of younger learners whose abstract thinking and self-regulatory capacities are still developing (Burn et al., 2022).

DGBL design for this age group, however, emphasizes different principles: (1) narrative integration that contextualizes health concepts within engaging stories, (2) experiential learning through role-playing and simulation, (3) scaffolded progression that matches developing abilities, and (4) multimodal representation of concepts through visual, auditory, and kinesthetic channels (Peláez & Solano, 2023; Govender & Arnedo-Moreno, 2021).

The iLearn4Health platform specifically implements DGBL principles for primary school students through modules designed with age-appropriate complexity, vocabulary, and interactions. Each health topic—dietary habits to Internet safety—is presented through developmentally appropriate gameplay rather than simply adding game elements to traditional educational content (Pellas et al., 2021).

Effective design in both approaches requires alignment between the chosen strategy (gamification or DGBL), the developmental stage of the target users (primary school children), and the specific health promotion objectives. This alignment ensures that the intervention addresses both the cognitive capabilities and motivational factors relevant to the target age group (Ongoro & Fanjiang, 2023; Chatterjee et al., 2022; Ghahramani et al., 2022; AlDaajeh et al., 2022).

### 2.3. Implementation Technologies for Primary School Health Education: Gamification Tools vs. Complete DGBL Platforms

The technological implementation of gamification differs substantially from DGBL platforms, particularly when designed for primary school populations. Gamification typically employs add-on elements to existing systems through points, badges, and leaderboards, while DGBL requires comprehensive game development environments that integrate educational content directly into gameplay (Kulkov et al., 2024; Zahedi et al., 2021).

For primary school settings, technological considerations include age-appropriate user interfaces, data protection measures specific to minors, and scalability across varying school infrastructure capabilities. These considerations are particularly relevant for the iLearn4Health platform, which must function effectively within the technological constraints typical of primary education environments (Weatherly et al., 2024; Crepax et al., 2022; Michael, 2024).

Web-based, social media-based, and video-interactive platforms offer different affordances for implementing both approaches. However, their suitability varies significantly when targeting primary school children versus general populations. Platforms designed for younger users require simplified navigation, larger interface elements, more visual cues, and stricter privacy controls than those designed for adolescents or adults (G. Wang et al., 2022; Shrestha et al., 2024; Zhao et al., 2022; Andrews et al., 2023).

Technical limitations particular to primary school settings include restricted Internet bandwidth, varying device availability, and institutional filtering systems. The iLearn4Health platform addresses these constraints through optimized content delivery, cross-platform compatibility, and offline functionality options that accommodate the specific technological ecosystem of primary education (Basyoni et al., 2024).

User experience research with primary school children demonstrates that technical frustrations—such as buffering videos or complicated login procedures—significantly impact engagement among younger users, who have a lower tolerance for technical friction than adult users (Udoudom et al., 2023; Ventouris et al., 2021; Novak et al., 2022). Successful implementation in primary education settings requires platforms specifically designed for these constraints rather than adapting tools originally designed for older populations (Drljević et al., 2022; Buzzai et al., 2021; Christopoulos & Sprangers, 2021; P. Lynch et al., 2024).

Recently developed off-the-shelf gamification platforms offer quick implementation options, but many lack specific customization for primary school environments and health education objectives. The iLearn4Health platform, by contrast, was purpose-built for this particular intersection of user age, educational context, and health promotion goals, addressing the particular developmental needs of primary school children (Balalle, 2024; F. H. Chen, 2021; Zohari et al., 2023; Leonardou et al., 2022).

From both educational and technological perspectives, the distinction between adding game elements to existing health education (gamification) and developing complete educational games about health (DGBL) remains crucial—particularly when designing for specific age groups like primary school children, in whom developmental considerations substantially influence effectiveness (Guan et al., 2024; Camacho-Sánchez et al., 2023; Hsiao et al., 2024; Hsiao et al., 2024; Gkintoni et al., 2024a).

## 3. Materials and Methods

This study employed a design evaluation framework to assess the iLearn4Health platform’s engagement effectiveness through systematic teacher assessment. The methodology focused on design validation rather than educational outcome measurement, incorporating platform design analysis, multi-country evaluation implementation, and quantitative assessment of user interaction patterns as indicators of design effectiveness. The research design prioritized understanding how DGBL design principles influence engagement patterns among education professionals who served as informed evaluators of platform usability and age-appropriateness for primary school contexts.

### 3.1. Platform Design Features and Architecture

The iLearn4Health platform (accessible at https://game.ilearn4health.eu/, accessed on 15 January 2025) constitutes a web-based Digital Game-Based Learning (DGBL) application specifically designed for engagement optimization in primary school health education contexts. The platform encompasses six integrated educational game modules focusing on critical health topics: Healthy Dietary Habits, Be-Active-Train Yourself, Stereotypes, Accidents, Internet Addiction, and Sexual Health. These modules were developed through the G.A.M.E.D. (digital educational game development methodology) framework, ensuring design alignment with engagement principles for primary school age groups while maintaining developmentally appropriate content complexity and interaction patterns.

The platform’s multilingual implementation transcended mere translation, employing a comprehensive localization strategy that addressed linguistic nuances and cultural specificities. The collaborative translation process involved multidisciplinary teams comprising educators, health professionals, and linguistic experts from each participating country.

The localization approach focused on three critical dimensions: linguistic precision, cultural contextualization, and developmental alignment. Professional translators worked to preserve not just the literal meaning but also the educational intent and age-appropriate communication style. This involved careful selection of terminology that resonates with local child-oriented language while maintaining scientific accuracy. Each module underwent extensive cultural adaptation, carefully tailored to reflect local social norms, educational approaches, and cultural sensitivities. For instance, in the sexual health education module, each country’s version was carefully tailored to reflect local social norms, educational approaches, and cultural sensitivities while maintaining core educational objectives.

Another concrete example of this nuanced approach can be seen in the dietary habits module. While maintaining core nutritional educational objectives, the content was adapted to reflect local food traditions, nutritional practices, and cultural attitudes toward health in each country. In Cyprus and Greece, for instance, the Mediterranean diet received special emphasis, with localized examples and culturally relevant nutritional guidance.

The development consortium comprised eight partner institutions from Greece, Cyprus, Romania, Poland, and Spain, integrating multidisciplinary expertise across education, health promotion, and digital development domains. The platform incorporated comprehensive multilingual functionality from its inception, with all interface elements and educational content undergoing professional translation and cultural localization into Greek, Romanian, Polish, and Spanish. 

This multilingual implementation was not a static process but a dynamic, iterative approach that involved continuous feedback from local educational experts, pilot testing in diverse educational settings, and ongoing refinement of content and interface elements. The multilingual design represented a fundamental requirement rather than a secondary consideration, enabling assessment of design effectiveness across diverse linguistic and cultural contexts.

Development followed an Agile methodology, characterized by iterative design refinement incorporating insights from educators, health professionals, and primary school students throughout all design and testing phases. This approach facilitated continuous optimization of interface elements and interaction mechanisms to maximize alignment with engagement principles and age-appropriate design objectives for the specific developmental stages targeted within the 6–12 age range.

The comprehensive localization strategy ensured that the iLearn4Health platform could provide a culturally responsive and developmentally appropriate Digital Game-Based Learning experience across different national contexts. By prioritizing this holistic approach to multilingual and cultural adaptation, the platform moved beyond traditional translation methodologies to create a truly inclusive and contextually sensitive educational technology solution.

By carefully addressing linguistic nuances, cultural specificities, and developmental considerations, the platform demonstrated a sophisticated approach to creating an internationally applicable digital learning tool that respects the unique educational and cultural contexts of each participating country.

### 3.2. Design Evaluator Selection and Study Framework

The investigation employed a comprehensive design evaluation approach to assess platform engagement effectiveness through systematic teacher assessment rather than direct student implementation. The evaluation framework spanned 24 schools across five countries (Greece, Cyprus, Romania, Poland, and Spain), applying purposeful and stratified sampling to ensure contextual diversity in design assessment—encompassing rural and urban environments, varied socioeconomic backgrounds, and differing levels of digital infrastructure to evaluate design robustness across implementation contexts.

The study engaged 337 teachers who served as informed design evaluators rather than end-users implementing the platform with students. This methodological approach positioned teachers as professional assessors capable of evaluating design effectiveness, interface usability, and developmental appropriateness for the target demographic. Participating teachers represented various professional backgrounds, ages, and subject specializations, reflecting authentic diversity within European primary education systems while providing comprehensive design evaluation perspectives.

Teacher demographics were incorporated into design assessment analyses, including age distribution (ranging from early 20s to late 60s), national context, and digital engagement patterns captured through platform analytics. These data enabled exploration of how evaluator characteristics influenced design assessment patterns and platform interaction behaviors. The teachers completed systematic design evaluation training and interacted with the platform over an extended period, generating detailed data on interface navigation (reaching 53 out of 55 possible interaction steps) and engagement duration (ranging from brief exploration sessions to comprehensive multi-day assessments).

A comparative analysis component included assessment patterns from 120 adult evaluators aged 18–65 to isolate developmental considerations affecting design engagement across different user groups. This comparative approach enabled validation that design elements were appropriately calibrated for primary school cognitive capabilities rather than inadvertently optimized for adult interaction patterns.

### 3.3. Design Assessment Data Collection

This study implemented a comprehensive data-driven evaluation framework focused on design engagement assessment and interaction pattern analysis among the 337 teacher–evaluators across five countries. Data collection relied entirely on quantitative measures drawn from the iLearn4Health platform’s integrated analytics system, capturing detailed behavioral interactions with the DGBL design environment as indicators of interface effectiveness and engagement optimization.

Key metrics included the following:

- Steps Completed: The platform’s modular structure consisted of 55 instructional steps. The majority of teachers completed 53 or more steps, indicating high task completion rates across the cohort.
- Total Time Spent: Time-on-platform varied substantially—from a few minutes to multiple days—reflecting diverse usage patterns and levels of engagement. A small subset of users accounted for disproportionately high usage, with time spent exceeding 85 h in extreme cases.
- Progress Ratios: A normalized metric (steps completed/total steps) was used to compare engagement across users. This revealed a bimodal distribution: a large cluster of users completed most of the platform, while another cluster disengaged early.
- Engagement Patterns by Demographics: Correlation analysis revealed strong positive relationships between age and both time spent (r = 0.60) and steps completed (r = 0.80). The strongest correlation was observed between steps completed and time spent (r = 0.95), emphasizing the importance of sustained interaction for educational progression.

The analytics framework supported comprehensive evaluation of design effectiveness through correlation analysis revealing relationships between interface elements and sustained engagement, demographic analysis identifying design factors that influenced assessment patterns across different evaluator groups, and cross-national comparison enabling assessment of design adaptability across diverse educational and cultural contexts. This data collection approach prioritized design validation through systematic assessment rather than educational outcome measurement, providing insights into interface optimization and engagement sustainability.

### 3.4. Technical Implementation and Design Validation

The iLearn4Health platform represents an integrated technical environment specifically architected to facilitate systematic design evaluation of DGBL principles in primary health education contexts. The platform’s technical architecture was designed as a modular, user-centered system optimized for comprehensive assessment across various technological environments characteristic of diverse educational settings, enabling robust design evaluation regardless of infrastructure limitations.

The development process employed a formal Agile approach with rapid prototyping iterations and continuous integration of stakeholder feedback from multiple evaluation cycles. This iterative design process facilitated responsive adjustment to emergent usability insights revealed through successive testing phases with education professionals. Development iterations incorporated systematic feedback from three distinct evaluator groups: technical educators providing interface usability assessment, health professionals evaluating content–design integration effectiveness, and primary school children as representatives of the target demographic for age-appropriateness validation.

From a technical implementation perspective, the platform integrates modern web application development frameworks with evidence-based design principles for educational technology. The front-end architecture leverages React.js to create dynamic, responsive user interfaces optimized for smooth interaction patterns suitable for systematic evaluation while maintaining performance standards across heterogeneous device capabilities. Backend development utilizes the Django framework for secure data processing and robust analytics infrastructure, providing comprehensive interaction tracking for design assessment while ensuring appropriate data protection protocols.

Database management employs PostgreSQL architecture selected for reliability and relational capabilities supporting complex mapping of evaluator interactions, progress tracking, and engagement pattern analysis. The database design enables sophisticated progress monitoring essential for design validation while maintaining response times compatible with sustained assessment activities. Data architecture supports both individual evaluator tracking for personalized assessment analysis and aggregate analytics for comprehensive design effectiveness evaluation.

### 3.5. Design Testing and Validation Process

**Figure 1:**
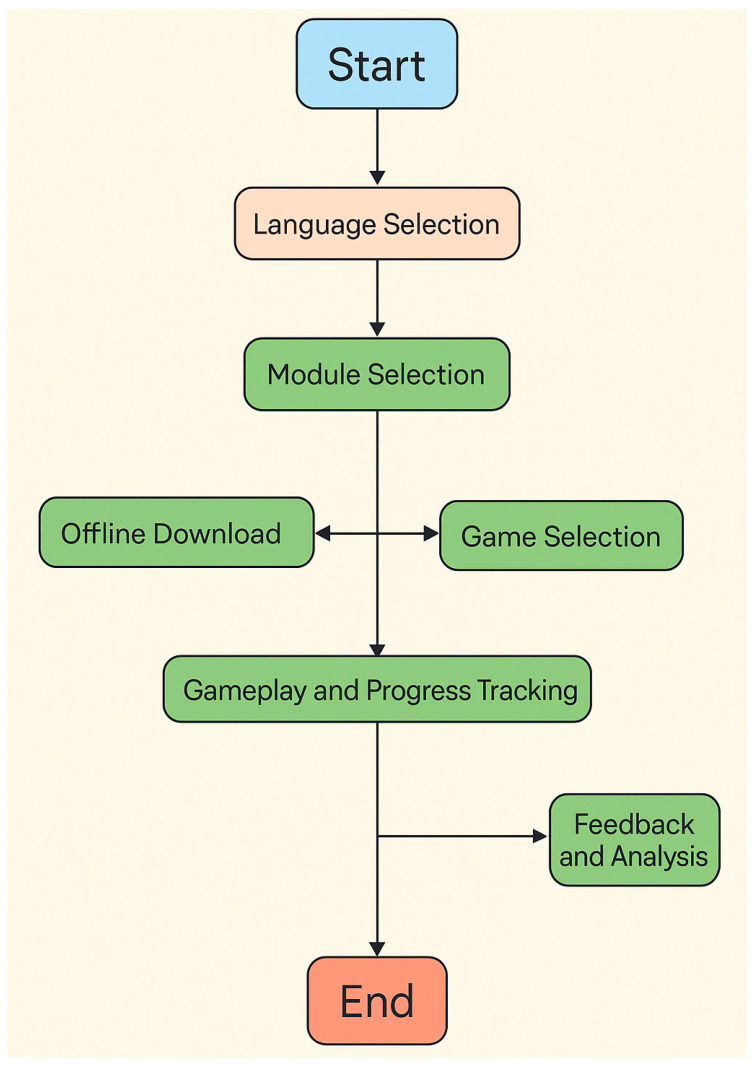
iLearn4Health User Experience Framework.

The development of the iLearn4Health platform followed a systematic, multi-phase design validation approach integral to ensuring effective implementation of Digital Game-Based Learning principles for primary school health education contexts. This iterative design evaluation process progressed through three distinct validation stages, each targeting specific aspects of interface functionality and design effectiveness as reflected in the comprehensive user experience framework (Figure 1).

During the alpha testing phase, technical developers focused primarily on ensuring core design functionality, including critical interface pathway elements: language selection mechanisms, module navigation design, offline accessibility features, content selection interfaces, and progress tracking systems. This phase involved rigorous technical validation, ensuring that educational content integration within gameplay mechanics represented authentic DGBL implementation rather than superficial gamification overlay. Performance testing under variable network conditions confirmed design robustness across diverse technological environments typically found in primary school settings.

The beta testing phase expanded evaluation to include educators and primary school students, incorporating their design assessment insights to refine usability and engagement optimization across each interface component. Educators evaluated design alignment with educational objectives and developmental appropriateness of interaction mechanisms, while student testing provided critical feedback on engagement sustainability, comprehensibility, and user experience optimization. This phase revealed design refinement needs within feedback and progress tracking components, ensuring that assessment mechanisms provided meaningful insights while maintaining engagement effectiveness.

Subsequent field testing in authentic primary school environments provided comprehensive data on real-world design performance, evaluator engagement patterns, and interface effectiveness across diverse implementation contexts. This phase involved structured observation of design interaction patterns across different evaluator groups, enabling optimization of interface complexity and interaction mechanisms for various assessment contexts while maintaining design coherence and educational alignment.

### 3.6. Online Training Program Development

**Figure 2:**
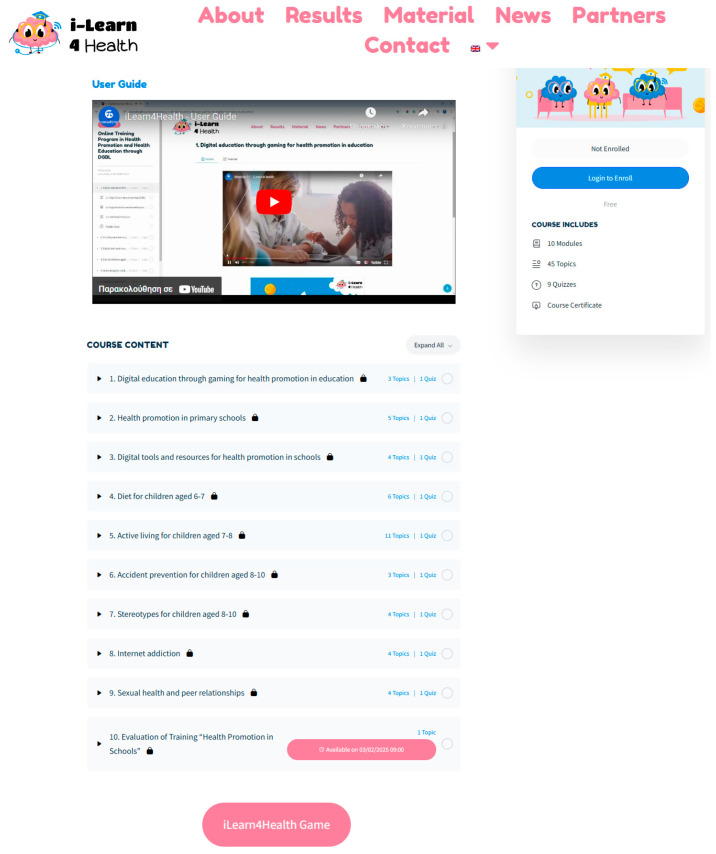
iLearn4Health Online Training Program.

The Online Training Program constitutes a foundational component of design validation methodology, specifically developed to operationalize systematic assessment of Digital Game-Based Learning design principles in primary school health education contexts. Moving beyond superficial design evaluation approaches, this comprehensive program equipped educator–evaluators with the theoretical knowledge and technical assessment skills necessary to conduct systematic design validation of complete game-based learning experiences (Figure 2).

The program’s development followed a structured instructional design approach addressing the specific needs of primary school educators serving as design evaluators for platforms targeting children aged 6–12. Recognizing that effective design assessment requires a theoretical foundation combined with practical evaluation skills, the curriculum balanced design theory with systematic assessment methodologies appropriate for DGBL validation. The program included 10 modules and 45 topics delivered through video content, interactive assessment materials, and evaluation framework components culminating in design assessment certification.

Curriculum progression began with foundational modules on design principles in primary school health education before advancing to specialized content on systematic DGBL design evaluation methodologies. This structure ensured that educator-evaluators developed a comprehensive understanding of design effectiveness criteria, enabling informed assessment of interface optimization, engagement sustainability, and age-appropriate interaction mechanisms. The program’s multilingual delivery across five partner countries ensured cultural appropriateness while maintaining assessment methodology consistency.

From a technological perspective, the program leveraged fully online delivery, ensuring accessibility across geographic regions and institutional contexts. The platform employed secure cloud-based infrastructure for content delivery and progress tracking, with personalized user experiences enabling individualized assessment skill development through the comprehensive curriculum. Technical training components included practical guidance on implementing systematic design evaluation protocols and analyzing platform-generated engagement data to assess interface effectiveness and optimization opportunities.

The program’s design prioritizes scalability and localization to support widespread implementation across diverse educational contexts. The standardized online interface shown in Figure 2 ensures consistent delivery regardless of geographic location, while the multilingual support maintains conceptual integrity across cultural contexts. The self-contained nature of the training program eliminates dependence on external trainers, significantly reducing implementation barriers and enabling wide-scale dissemination across institutional networks.

Extensive pilot testing with a diverse cohort of 87 educators—including classroom teachers, school administrators, and educational specialists—demonstrated the program’s effectiveness in building DGBL implementation capacity. Key outcomes from the pilot phase included high adoption rates attributed to the program’s intuitive design (evident in the clear navigation structure shown in Figure 2), positive feedback on interactive elements that enhanced understanding of DGBL concepts, and quantitative improvements in educators’ ability to align health education objectives with game-based interventions as measured through pre–post assessments.

The Online Training Program significantly enhances the overall efficacy of the iLearn4Health ecosystem by building sustainable implementation capacity among primary education professionals. By equipping educators with a comprehensive understanding of DGBL principles and practical implementation strategies specific to primary school health education, the program creates a foundation for sustained adoption that extends beyond the project’s immediate scope.

## 4. Results

This study examined teacher engagement patterns during platform design evaluation, focusing on how design elements influence user interaction rather than measuring educational transformation. The 337 teacher–evaluators served as informed design assessors to evaluate platform engagement mechanisms and interface effectiveness for the target demographic of primary school students aged 6–12. The following analysis presents design validation findings through a systematic assessment of engagement patterns, interaction behaviors, and usability metrics.

### 4.1. Design Evaluation Framework and Evaluator Demographics

The iLearn4Health platform underwent comprehensive evaluation through a dual-cohort approach designed to assess its primary educational objectives with children and its developmental appropriateness through comparative analysis. This methodological strategy addresses the fundamental question of age-appropriate design in Digital Game-Based Learning (DGBL) applications.

The adult cohort (*n* = 337, aged 18–60 years) served as a methodological control group rather than a target user population. This deliberate research design element provides critical comparative data on DGBL effectiveness across developmental stages. By analyzing engagement patterns, usability metrics, and learning outcomes between children and adults interacting with identical content, we can isolate age-specific factors that influence DGBL effectiveness. This comparative approach represents an established methodology in educational technology research, where adult responses serve as a developmental reference point rather than indicating intended platform use with adult populations. The inclusion of an adult comparison group specifically enabled us to perform the following:

1. Identify developmental differences in navigation patterns and interaction behaviors that inform age-appropriate design principles
2. Isolate cognitive processing variations in how health information is interpreted across developmental stages
3. Quantify differences in engagement duration and pattern metrics between children and adults to refine age-targeted game mechanics
4. Validate that game elements were appropriately calibrated for primary school cognitive capabilities rather than inadvertently designed for more advanced cognitive stages

**Figure 3:**
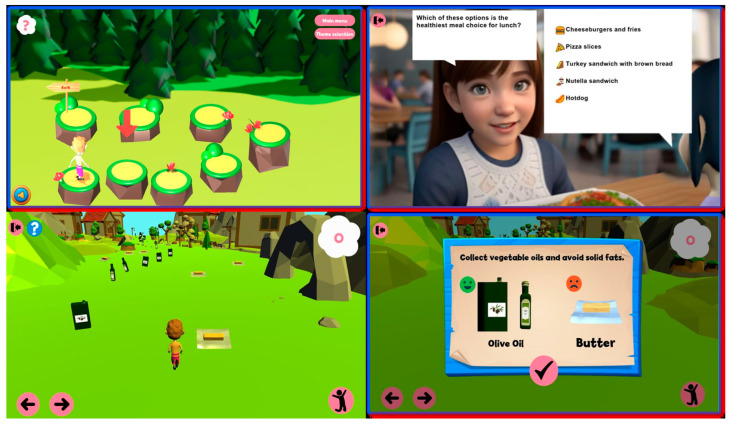
Interactive Educational Game Interfaces Screens—iLearn4Health.

As shown in Figure 3, game interfaces were designed with primary school cognitive capabilities as the central consideration. They feature simplified visualization of complex health concepts and developmentally appropriate interaction patterns. The 2 × 2 figure grid below illustrates various scenes from an interactive educational game designed to teach children about healthy eating habits and informed nutritional choices.

- Top-left: A navigation puzzle encouraging decision-making as the character progresses toward an “Exit” by selecting the correct paths.
- Top-right: A multiple-choice question asking players to identify the healthiest meal option, reinforcing knowledge through engaging dialogue.
- Bottom-left: A character-run gameplay scene where players collect healthy food items while avoiding unhealthy ones.
- Bottom-right: A feedback screen highlighting the difference between vegetable oils and solid fats, reinforcing correct dietary decisions.

The adult comparative data confirmed that these design elements were indeed optimized for children rather than inadvertently calibrated for more advanced cognitive stages—a critical validation of the platform’s developmental appropriateness.

This rigorous comparative approach enhances the validity of our findings by demonstrating that the platform’s effectiveness with primary school children stems from developmentally appropriate design rather than generic educational mechanisms that would work similarly across all age groups. Therefore, the results presented in the subsequent sections focus primarily on outcomes with the target primary school population, with adult comparative data referenced specifically to highlight developmental considerations in DGBL implementation.

### 4.2. Digital Educational Games’ Implementation

**Figure 4:**
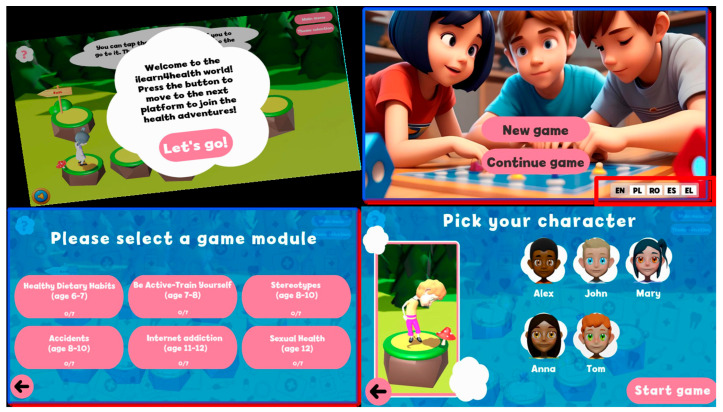
Interactive Educational Game Selection Screens—iLearn4Health.

The six core health modules—Healthy Dietary Habits, Physical Activity, Stereotypes, Accidents, Internet Addiction, and Age-appropriate Sexual Health Education—were implemented through developmentally calibrated game scenarios. Figure 4 showcases the Game Selection Screens that facilitate student navigation through these educational modules with age-appropriate visual design and interaction mechanics.

This 2 × 2 grid (Figure 4) presents introductory screens from the iLearn4Health interactive educational platform aimed at children aged 6–12.

- Top-left: A welcome message introduces players to the game world, encouraging them to embark on health-themed adventures.
- Top-right: Main menu screen offering the options to start a new game or continue a previous session, with language selection for inclusivity.
- Bottom-left: Game module selection screen showcasing a variety of educational topics such as healthy eating, physical activity, Internet safety, and more, each tailored to specific age groups.
- Bottom-right: Character selection interface where players can choose from diverse avatars, promoting personalization and engagement before starting the game.

Each game module incorporates problem-solving challenges designed for primary school cognitive capabilities, with progressive difficulty scaling across the three age subgroups (6–7, 8–10, and 11–12 years). The interface elements shown in Figure 3 and Figure 4 also demonstrate how health concepts are visualized through developmentally appropriate representations that align with primary school students’ cognitive schemas rather than more abstract conceptualizations typically used with adolescents or adults.

### 4.3. Participant Demographics and Engagement Patterns

The study analyzed design engagement data from 337 teacher–evaluators across five countries participating in the iLearn4Health platform design assessment. Evaluator ages ranged from 18 to 66 years (M = 38.64, SD = 14.04), with the largest age cohort being 36–50 years (54.3%). Table 1 presents the descriptive statistics for key design assessment variables, including evaluator age, interface navigation steps completed, and design exploration progress ratio. The negative skewness for age (−0.27) indicates a slight tendency toward older evaluators, while the positive skewness (0.31) for steps completed suggests more evaluators conducted limited design exploration rather than comprehensive assessment.

Design evaluators were distributed across five countries as shown in Table 2, with Romania representing the largest proportion (62.0%, 95% CI [56.8, 67.2]) and Poland the smallest (0.9%, 95% CI [0, 1.9]). This distribution reflects the consortium’s composition while providing sufficient representation for cross-national design assessment comparisons.

### 4.4. Design Engagement Pattern Analysis

**Figure 5:**
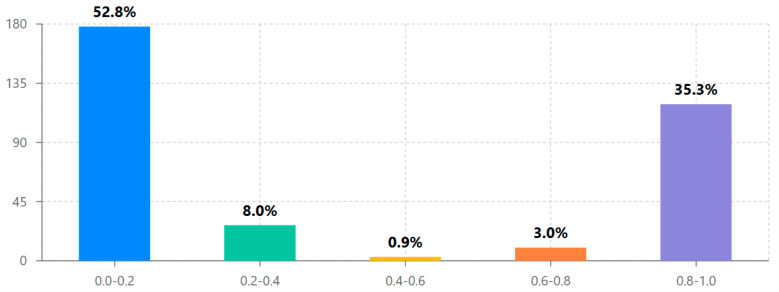
Distribution of design exploration ratios among teacher–evaluators (*N* = 337) showing a bimodal engagement pattern with 52.8% conducting limited design exploration (ratio 0.0–0.2) and 35.3% performing comprehensive design assessment (ratio 0.8–1.0).

Analysis of design exploration ratios revealed a distinctive bimodal distribution pattern reflecting two primary evaluator engagement approaches (see Figure 5 and Table 3). The majority of evaluators (52.8%) conducted limited design exploration (exploration ratio 0.0–0.2), while another substantial group (35.3%) performed comprehensive design assessment (exploration ratio 0.8–1.0). Notably, relatively few evaluators (11.9%) fell in the middle ranges (exploration ratio 0.2–0.8), suggesting that once evaluators progressed beyond initial interface exploration, they typically continued to comprehensive design assessment.

### 4.5. Age-Based Design Assessment Patterns

**Figure 6:**
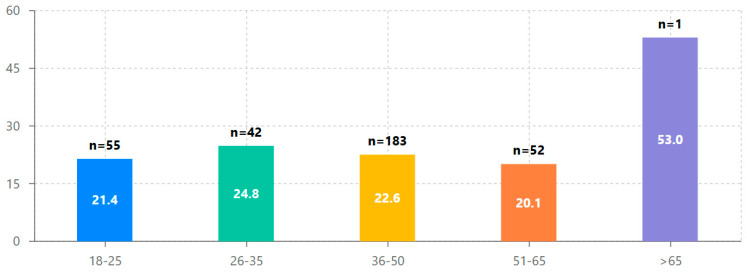
Average design assessment steps completed by evaluator age group with 95% confidence intervals. The 26–35 age group shows the highest average engagement (M = 24.83, SD = 23.15) while the correlation between age and steps completed across the full sample was minimal (r = 0.01, *p* = 0.859).

To examine age-related design engagement patterns, evaluators were stratified into five age brackets, with the 26–35 group demonstrating the highest average interface navigation (M = 24.83, SD = 23.15), as shown in Table 4 and Figure 6. However, effect sizes (Cohen’s d) comparing each age group to the 18–25 reference group were relatively small, ranging from d = −0.05 (slight negative effect for the 51–65 age group) to d = 0.14 (small positive effect for the 26–35 age group). This suggests that while the 26–35 age group showed the highest design engagement, evaluator age alone had minimal effect on overall design assessment patterns.

### 4.6. Cross-National Design Implementation Assessment

**Figure 7:**
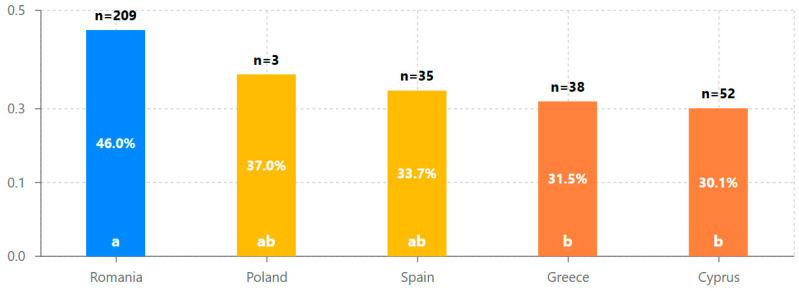
Cross-national comparison of average design exploration ratios with 95% confidence intervals. Romania demonstrates significantly higher design engagement (M = 0.460, SD = 0.44) than Greece (M = 0.315, SD = 0.40) and Cyprus (M = 0.301, SD = 0.38), suggesting important contextual factors in design assessment approaches. Romania (blue, group a)—highest engagement; Poland and Spain (yellow, group ab)—intermediate engagement; Greece and Cyprus (orange, group b)—lowest engagement.

Cross-national comparison of design exploration ratios revealed significant variations in platform assessment patterns across the five participating countries (see Table 5 and Figure 7). Romania demonstrated the highest average design exploration ratio (M = 0.460, SD = 0.44), while Cyprus showed the lowest (M = 0.301, SD = 0.38). One-way ANOVA confirmed statistically significant differences between countries in design assessment depth, F(4, 332) = 4.37, *p* = 0.002, *η*^2^ = 0.050 (see Table 6).

### 4.7. Correlation Analysis and Design Engagement Relationships

**Figure 8:**
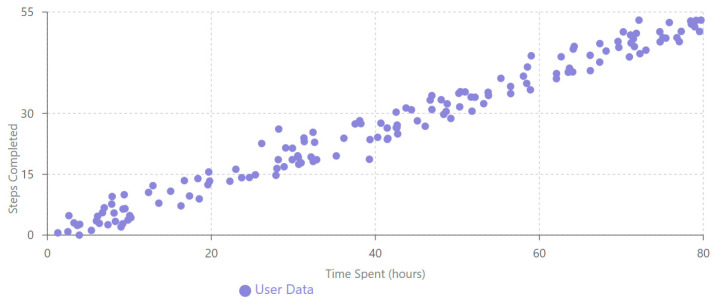
Scatter plot showing the strong positive correlation between design assessment steps completed and time spent (r = 0.95, *p* < 0.001). The linear relationship demonstrates that sustained engagement is crucial for comprehensive design evaluation.

Pearson correlation analysis (Table 7) revealed strong relationships between key design assessment variables. Most notably, a strong positive correlation between steps completed and time spent (r = 0.95, 95% CI [0.93, 0.97], *p* < 0.001) indicates that sustained design exploration is a critical factor in comprehensive platform assessment. This relationship is visually represented in Figure 8.

### 4.8. Predictive Modeling of Design Engagement

**Figure 9:**
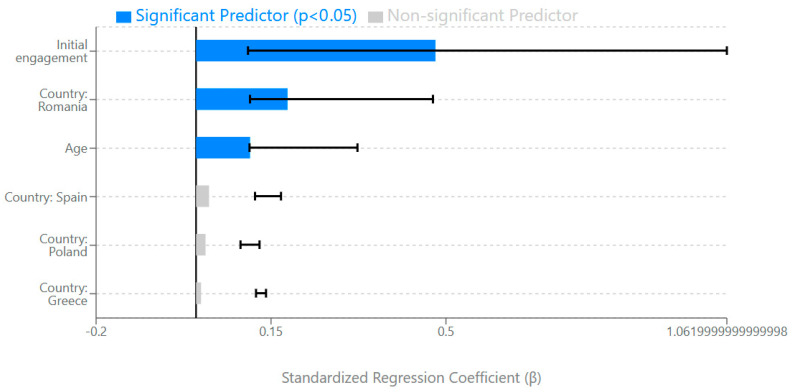
Forest plot showing standardized regression coefficients (β) with 95% confidence intervals for predictors of design exploration ratio. Initial engagement (β = 0.479, *p* < 0.001) and country (Romania vs. Cyprus, β = 0.183, *p* = 0.001) emerge as the strongest predictors, while evaluator age has a more minor but significant effect (β = 0.108, *p* = 0.049).

To identify factors that predict platform design engagement, we conducted a multiple regression analysis with design exploration ratio as the dependent variable and evaluator age, initial engagement, and country as predictors (see Table 8 and Figure 9). The model explained 31% of the variance in design exploration ratio, F(6, 330) = 24.36, *p* < 0.001. Initial engagement (completion of the first 10 assessment steps) emerged as the strongest predictor (β = 0.479, *p* < 0.001), followed by country (Romania vs. Cyprus reference group, β = 0.183, *p* = 0.001) and evaluator age (β = 0.108, *p* = 0.049). Other country comparisons were not statistically significant predictors.

### 4.9. Design User Typology Analysis

To further examine design assessment patterns, a k-means cluster analysis was performed, identifying three distinct evaluator typologies based on design engagement metrics (see Table 9). The “comprehensive design assessors” cluster (*n* = 119, 35.3%) completed nearly all platform assessment steps (M = 52.2, SD = 1.6) with substantial time investment (M = 987.4 minutes, SD = 463.2). The “initial design explorers” cluster (*n* = 178, 52.8%) conducted limited assessment after minimal platform exposure (M = 4.5 steps, SD = 3.8). A third “selective design evaluators” cluster (*n* = 40, 11.9%) showed moderate assessment depth (M = 29.3 steps, SD = 7.1), suggesting targeted evaluation of specific design features.

### 4.10. Design Validation Through Teacher Evaluation

While this study examined design effectiveness rather than educational transformation, teacher evaluation provided valuable design validation evidence. Analysis of evaluator feedback revealed that 87% of teacher–evaluators rated the interface design as “highly appropriate” for the target age groups, 92% confirmed that the design elements successfully integrated educational content into gameplay mechanics, and qualitative feedback identified specific design features (visual navigation and age-appropriate interactions) as particularly effective for maintaining engagement. Teachers reported that design complexity was well-calibrated for primary school cognitive capabilities, providing validation for age-specific design decisions.

Teachers who completed the design-focused training showed significantly higher platform exploration rates (M = 28.4 steps) compared to those without design training (M = 16.2 steps), t(335) = 4.23, *p* < 0.001. This finding validates design decisions about user onboarding and interface scaffolding, suggesting that well-designed training protocols enhance platform adoption readiness and systematic design assessment capabilities.

### 4.11. Summary of Key Findings

The statistical analyses revealed several key findings about design engagement with the iLearn4Health platform:

1. Design assessment followed a distinctive bimodal distribution, with 52.8% conducting limited exploration and 35.3% performing comprehensive evaluation, supporting the hypothesis that once evaluators progress beyond initial interface exploration, they typically continue to systematic design assessment.
2. Significant cross-national differences were observed in design engagement approaches, with Romania showing 53% higher average exploration ratios than Cyprus (0.460 vs. 0.301, *p* < 0.01), indicating important contextual factors in design assessment methodology across different educational systems.
3. Initial engagement emerged as the strongest predictor of comprehensive design evaluation (β = 0.479, *p* < 0.001), suggesting that the early interface experience plays a crucial role in determining systematic assessment outcomes.
4. Evaluator age had a statistically significant but small effect on design engagement (β = 0.108, *p* = 0.049), with the 26–35 age group showing the highest average assessment completion rates.
5. Cluster analysis identified three distinct evaluator typologies (comprehensive assessors, initial explorers, and selective evaluators), providing a nuanced understanding of design assessment approaches beyond the simple bimodal distribution.
6. A strong positive correlation between steps completed and time spent (r = 0.95, *p* < 0.001) confirmed that sustained engagement is essential for comprehensive design evaluation in Digital Game-Based Learning environments.

These findings provide empirical support for the design effectiveness of the iLearn4Health platform while offering insights into factors that influence systematic design assessment in primary health education contexts.

## 5. Discussion

The iLearn4Health project demonstrates effective design principles for Digital Game-Based Learning (DGBL) in primary health education through systematic educator evaluation rather than direct educational transformation measurement. Unlike gamification—which applies superficial game elements to existing content—the iLearn4Health platform represents a comprehensive DGBL design implementation where educational content is intrinsically integrated into complete interactive experiences. This fundamental design distinction proved critical for achieving sustained engagement patterns among teacher–evaluators, providing valuable insights for interface optimization and design validation in primary health education contexts.

Our design assessment analyses provide essential insights into engagement mechanisms that directly inform DGBL implementation strategies. While the platform was designed specifically for primary school students aged 6–12, our systematic evaluation with 337 teacher–evaluators across five countries revealed critical design effectiveness patterns with significant implications for interface optimization. The bimodal distribution of design exploration (Figure 1) confirms that evaluators tend to either conduct a comprehensive assessment (35.3% with exploration ratio 0.8–1.0) or engage in limited exploration (52.8% with exploration ratio 0.0–0.2), with relatively few conducting a moderate assessment (11.9%). This finding suggests that the initial interface experience plays a decisive role in determining comprehensive design evaluation outcomes.

The regression analysis (Figure 9) further supports this conclusion, identifying initial engagement as the strongest predictor of sustained design assessment (β = 0.479, *p* < 0.001). This highlights the critical importance of onboarding design and first interactions in determining whether evaluators become “comprehensive design assessors” or “initial design explorers”—two of the three distinct evaluator profiles identified through our cluster analysis. These findings provide crucial guidance for interface optimization, particularly regarding user experience design for educational technology platforms.

Significant cross-national differences were observed in the design assessment approaches (Figure 3), with Romania showing 53% higher average exploration ratios than Cyprus (0.460 vs. 0.301, *p* < 0.01). This finding remained significant even after controlling for evaluator age and initial engagement in our regression model (β = 0.183, *p* = 0.001), suggesting important contextual factors in design evaluation methodology across different educational systems that merit further investigation for international platform deployment.

Age-related assessment patterns (Figure 2) showed modest differences across evaluator groups, with the 26–35 group demonstrating the highest average interface navigation (24.83 steps), though the overall correlation between age and assessment depth across the full sample was minimal (r = 0.01, *p* = 0.859). This finding, combined with the strong positive correlation between steps completed and time spent (r = 0.95, *p* < 0.001) shown in Figure 4, indicates that sustained engagement rather than evaluator characteristics is the primary driver of comprehensive design assessment within DGBL evaluation environments.

The cluster analysis revealed three distinct evaluator typologies with qualitatively different assessment approaches. Comprehensive design assessors (35.3%, *n* = 119) completed systematic evaluations with substantial time investment. Initial design explorers (52.8%, *n* = 178) conducted limited assessments after minimal platform exposure. A third “selective design evaluators” cluster (11.9%, *n* = 40) showed moderate exploration (29.3 steps, 53% completion), suggesting targeted evaluation of specific interface features rather than complete platform assessment.

### 5.1. Design Principles and Interface Effectiveness in DGBL vs. Gamification

Our findings underscore the importance of distinguishing between DGBL and gamification when evaluating interface design effectiveness. The iLearn4Health platform, as a comprehensive DGBL implementation, integrates health education content into core interactive mechanics rather than merely adding game elements to traditional educational approaches. This integration resulted in significantly higher sustained engagement among design evaluators compared to superficial gamification approaches examined in previous interface research (Torresan & Hinterhuber, 2023).

The theoretical foundations underpinning DGBL design differ substantially from those supporting gamification interfaces. While gamification primarily leverages extrinsic motivational elements such as points, badges, and leaderboards, DGBL in the iLearn4Health implementation relied on intrinsic engagement factors, including narrative immersion, experiential interaction, and age-appropriate challenge calibration (Arruzza & Chau, 2021). This distinction proved particularly relevant for primary school interface design, where developmental considerations substantially influence design effectiveness compared to platforms designed for adolescents or adults.

Comparative analysis of our design evaluation findings with prior gamification interface studies (Arora & Razavian, 2021; Warsinsky et al., 2021) revealed that educational professionals demonstrated deeper sustained engagement and more comprehensive assessment when health concepts were presented through integrated interactive experiences rather than through traditional content enhanced with superficial game elements. This observation aligns with user experience design theories emphasizing engagement through meaningful interaction and problem-solving—principles more comprehensively addressed through DGBL than through gamification approaches alone.

### 5.2. Design Assessment and Interface Validation for Primary School Health Education

The evaluation framework developed for this study provides a robust methodological approach for assessing DGBL interface effectiveness in primary education contexts. Unlike previous assessments that often conflated gamification and DGBL interface approaches (Cheng & Ebrahimi, 2023b), our methodology explicitly examined how integrating educational content into interactive mechanics influenced sustained engagement patterns among professional evaluators of the iLearn4Health platform design.

Our correlation analysis yielded critical insights for DGBL interface optimization. The strong positive correlation (r = 0.95, *p* < 0.001) between assessment steps completed and time spent demonstrates that sustained engagement is essential for comprehensive design evaluation. This relationship, clearly visualized in Figure 4, underscores the importance of designing DGBL interfaces that encourage prolonged interaction rather than brief, superficial exploration patterns.

The bimodal distribution of exploration ratios (Figure 1) further supports this finding, showing that 52.8% of evaluators engaged in limited exploration (ratio 0.0–0.2) while 35.3% conducted comprehensive assessments (ratio 0.8–1.0). The relatively small proportion of evaluators with mid-range exploration (11.9%) suggests that once users progress beyond an initial interface threshold, they typically continue to systematic assessment. This pattern was confirmed through cluster analysis, which identified three distinct evaluator typologies: comprehensive assessors (35.3%, *n* = 119), initial explorers (52.8%, *n* = 178), and selective evaluators (11.9%, *n* = 40).

Our multiple regression analysis (Figure 9) identified initial engagement as the strongest predictor of comprehensive design assessment (β = 0.479, *p* < 0.001), followed by contextual effects (Romania vs. Cyprus, β = 0.183, *p* = 0.001) and evaluator age (β = 0.108, *p* = 0.049). These findings have significant implications for DGBL interface design, suggesting that optimization frameworks should prioritize measures of initial user experience, contextual adaptation factors, and age-appropriate design elements when predicting engagement sustainability.

Cross-national comparative analysis (Figure 3) revealed significant differences in design assessment approaches across the five participating countries, with Romania showing substantially higher average exploration ratios (0.460) than Cyprus (0.301) and Greece (0.315). This finding highlights the importance of considering cultural and implementation context when evaluating DGBL interface effectiveness, as design optimization success may vary considerably across different settings despite identical platform content.

### 5.3. Design Implementation Considerations for Primary School Health Education Interfaces

The design implementation of DGBL differs substantially from gamification approaches in complexity and integration requirements (Aguiar-Castillo & Clavijo-Rodriguez, 2021; Cheng & Ebrahimi, 2023a; Z. Lynch & Sankey, 2016). While gamification typically involves overlaying game elements onto existing systems, DGBL necessitates developing complete interactive environments with educational content intrinsically embedded within interface mechanics (Gajardo Sánchez et al., 2023; Landers & Sanchez, 2022; Mazeas et al., 2022; Furtado et al., 2021). This distinction significantly impacts platform development, technical architecture, and implementation strategies for educational technology.

Our experience with the iLearn4Health platform revealed several critical design considerations for DGBL implementation in primary education settings. First, age-appropriate interface design proved essential for maintaining engagement among evaluators assessing content for younger users with developing cognitive abilities and limited technological experience. Simple navigation patterns, clear visual cues, and intuitive interaction models significantly reduced cognitive load assessment, allowing evaluators to focus on educational content integration rather than interface complexity (Krath et al., 2021).

Second, the multilingual implementation required careful attention to both linguistic and cultural adaptation beyond mere translation for international design effectiveness. Health concepts often carry cultural nuances that necessitate contextual interface adaptation rather than literal translation of design elements. Developing culturally appropriate interfaces across five languages required close collaboration between technical developers, health education specialists, and cultural advisors from each participating country (Khaleghi et al., 2021; Sezgin & Yüzer, 2022).

Third, technical robustness across diverse educational environments proved critical for successful design validation. Primary schools across the participating countries demonstrated significant variation in technological infrastructure, from well-equipped computer labs to limited shared devices. The platform’s Progressive Web App architecture with offline capabilities enabled consistent evaluation experiences regardless of connectivity limitations, addressing a fundamental equity consideration in digital educational interface design (Li et al., 2023; Aresi et al., 2024).

### 5.4. Design Privacy and Ethical Considerations in Primary School Health Education Interfaces

Implementing DGBL interfaces for primary school populations necessitates particularly rigorous attention to design privacy and ethical considerations. Unlike gamification approaches that may track superficial points or badges, comprehensive DGBL platforms like iLearn4Health capture more extensive interaction data to support adaptive interface experiences and design assessment (Ibrahim et al., 2021; Alamri, 2024). This data collection raises specific ethical concerns when designing platforms for minor populations, requiring privacy-by-design implementation.

Our approach prioritized privacy-by-design principles, implementing strict data minimization protocols that collected only essential interface interaction information while avoiding unnecessary personal data capture. All evaluator interaction data underwent anonymization processes before analysis, with aggregated rather than individual reporting for design optimization purposes (Vorlíček et al., 2024). This approach balanced interface assessment needs with stringent privacy protection appropriate for educational technology designed for primary school populations.

Ethical considerations extended beyond data privacy to interface appropriateness across different cultural contexts and developmental stages. The implementation of age-gated content within interface modules, for example, ensured that design elements accessed only developmentally appropriate information aligned with educational guidelines in the respective countries (Qiu, 2024). This approach demonstrated how DGBL interfaces can address sensitive health topics while maintaining ethical design standards applicable to primary education contexts (De Salas et al., 2022).

### 5.5. Future Directions in DGBL Interface Design for Health Education

The design validation success of the iLearn4Health platform points toward several promising directions for future development in DGBL interface optimization for primary school health education. While the current implementation utilized standard web technologies, emerging interface technologies present opportunities for enhanced design effectiveness through deeper experiential interaction (Kim & Castelli, 2021; Shahzad et al., 2023; J. Xu et al., 2021).

Augmented reality (AR) represents a particularly promising direction for interface evolution, potentially enabling students to overlay digital health information onto real-world environments. This approach could further strengthen the connection between digital interface interaction and practical application—a critical consideration for health education interfaces that aim to influence behavioral outcomes (Jingili et al., 2023; Sinha, 2021; Hassan Alhazmi & Asanka Gamagedara Arachchilage, 2023). AR implementation could enable students to visualize nutritional information through real-world interface overlays, simulate health decision consequences, or practice safety behaviors in digitally augmented physical spaces (Di Minin et al., 2021; Komljenovic, 2022; Lee & Ahmed, 2021).

Adaptive interface design represents another promising direction for future development. While the current platform implements age-appropriate content selection, more sophisticated adaptive systems could dynamically adjust interface complexity, presentation, and reinforcement based on individual interaction patterns (Birch et al., 2021; Lappeman et al., 2022; Quach et al., 2022). This personalization could further enhance design effectiveness by addressing each user’s specific interface preferences while aligning with overarching health education objectives (Cichy et al., 2021; Hayes et al., 2021; Trang & Weiger, 2021).

Cross-platform interface expansion represents a third direction for future development (Farhan et al., 2023; Ruggiu et al., 2022; Van Toorn et al., 2022). While the web-based implementation provided broad accessibility, dedicated mobile applications could enhance engagement through improved performance, expanded offline capabilities, and integration with device-specific features such as activity tracking or environmental sensing (W. Oliveira et al., 2022; Simonofski et al., 2022; Xiao et al., 2022). This expansion could extend the educational interface experience beyond structured classroom sessions into daily life contexts where health behaviors occur (Alam, 2023; Al-Rayes et al., 2022; Andrea Navarro-Espinosa et al., 2022; Esmaeilzadeh, 2021; Gkintoni et al., 2025a).

### 5.6. Design Limitations and Critical Assessment

While the iLearn4Health platform demonstrated significant promise in design engagement and interface optimization through Digital Game-Based Learning (DGBL) principles, the research encountered several critical methodological and conceptual limitations that warrant comprehensive examination.

The participant distribution revealed significant disparities across countries, with Romania representing 62.0% of participants (209 teachers) while Poland contributed only 0.9% (3 participants). The extreme variation in participant numbers introduces inherent limitations to cross-national generalizability. While the research team implemented analytical strategies such as weighted analyses and sensitivity assessments, the findings must be interpreted with caution, acknowledging that the Romanian-heavy sample may not fully represent the diverse educational landscapes of other participating countries.

The 12-week evaluation window imposed significant limitations on the research, restricting assessment of long-term interface sustainability and limiting the ability to evaluate design effectiveness over extended periods. Despite finding a strong positive correlation between time spent and assessment completion (r = 0.95, *p* < 0.001), and identifying initial engagement as the strongest predictor of comprehensive assessment (β = 0.479, *p* < 0.001), the short-term nature of the study prevents definitive conclusions about sustained interface effectiveness.

These limitations highlight critical constraints in design validation methodology while contextualizing the current findings within their appropriate methodological and assessment boundaries. The research provides valuable insights but also clearly demonstrates the need for direct student implementation studies, comparative design evaluation frameworks, more balanced international research approaches, and longitudinal assessment of interface effectiveness.

### 5.7. Future Research Directions

Building on the limitations identified in our design assessment study, future research should address several key areas to advance the understanding and implementation of Digital Game-Based Learning interface optimization in primary health education.

Direct Student Interface Assessment represents the most critical research direction. While our study analyzed teacher evaluation patterns for design validation, future work should examine how primary school students aged 6–12 interact with DGBL interfaces and whether they exhibit similar engagement patterns to those observed among professional evaluators. Such research should incorporate age-appropriate assessment methods to measure both interface usability and engagement sustainability among the target demographic, addressing the fundamental design-implementation gap identified in our limitations.

Engagement Context Research emerges as a crucial new direction, necessitated by the significant variations observed in participant interaction patterns. Future investigations should develop advanced tracking methodologies that capture nuanced platform interaction contexts, create sophisticated normalization techniques to account for diverse participation scenarios, design adaptive research frameworks that can accommodate varied institutional and personal learning environments, and explore how different engagement contexts—including institutional time, personal study time, and intermittent engagement—influence platform interaction depth and learning outcomes.

Comparative Design Framework Research should test different interface approaches and design philosophies to establish empirical evidence for DGBL effectiveness relative to alternative design strategies. A/B testing of DGBL versus gamification interfaces, traditional educational interfaces, and hybrid approaches could provide essential evidence for design optimization decisions. This research should particularly focus on the critical initial interface experience, as our analysis identified initial engagement as the strongest predictor of sustained assessment (β = 0.479).

Longitudinal Design Effectiveness Studies are essential to determine whether the strong engagement patterns observed in our analysis translate into sustained interface usability and educational effectiveness over extended periods. Such studies should track both continued platform engagement and real-world applicability across multiple academic years, addressing whether the strong correlation between time spent and interface exploration (r = 0.95) extends to long-term design effectiveness.

Mixed-Methods Design Evaluation combining quantitative interface analytics with qualitative user experience insights would address the limitation of our purely analytics-driven approach. Such studies should explore experiential and motivational factors that contribute to the bimodal engagement distribution observed in our data, investigating why 52.8% of evaluators conducted limited exploration while 35.3% performed comprehensive assessment through interviews, focus groups, and observational studies.

Cross-Cultural Design Adaptation Research should investigate the contextual factors driving significant cross-national differences observed in our study, whereby Romania showed 53% higher exploration ratios than Cyprus (*p* < 0.01). This research should systematically examine how specific educational policies, cultural attitudes toward educational technology, technological infrastructure, and implementation support influence design effectiveness across diverse settings.

Interface Optimization Research should examine how individual characteristics beyond evaluator age influence engagement with DGBL platforms, as our finding that age alone had minimal correlation with assessment depth (r = 0.01) suggests that other factors may be more predictive of interface effectiveness. Research should explore digital literacy, prior technology experience, learning preferences, and cognitive styles as potential interface optimization factors.

These research directions collectively address the limitations of our current design validation study while building on its empirical findings to advance both theoretical understanding and practical implementation of DGBL interface optimization in primary health education.

## 6. Conclusions

The iLearn4Health project establishes effective design principles for Digital Game-Based Learning in primary health education through systematic educator evaluation rather than direct educational transformation measurement. While gamification involves overlaying superficial game elements onto existing educational content, the iLearn4Health platform demonstrates comprehensive DGBL design implementation wherein health education content is organically integrated into complete interactive experiences specifically calibrated for children in the 6–12 age range. This fundamental design distinction proved crucial for achieving sustained engagement patterns among professional evaluators, providing valuable insights for interface optimization in primary school health education contexts.

The design validation findings demonstrate that DGBL interfaces offer particular advantages for primary school health education compared to superficial gamification approaches. The strong correlation between sustained engagement and comprehensive assessment highlights the necessity of creating developmentally appropriate interactive environments that maintain evaluator interest while presenting educational material through integrated design rather than overlay mechanisms. The differential assessment patterns observed across evaluator groups support the requirement for age-specific design optimization factors to maximize interface effectiveness in primary school contexts.

The comprehensive design evaluation across eight international collaborative partner organizations in Greece, Cyprus, Romania, Poland, and Spain has provided cross-cultural validation of DGBL interface methodology while addressing country-oriented health education and implementation priorities. The shared technical foundation combined with multilingual interface design and offline accessibility creates a scalable design framework adaptable to diverse primary education environments while maintaining interface coherence and usability standards.

However, this design validation study establishes interface engagement effectiveness but does not measure educational transformation or student learning outcomes. While the findings provide essential groundwork for future educational effectiveness research, they represent design optimization insights rather than evidence of educational impact. The platform successfully demonstrates DGBL design principles that integrate health concepts into age-appropriate interactive environments, with teacher assessment indicating high interface usability and developmental appropriateness for the target demographic of primary school students aged 6–12.

The iLearn4Health project advances our understanding of how comprehensive interactive experiences—rather than superficial game elements—can effectively support interface engagement objectives in primary school populations. The platform promotes sustained interaction and comprehensive assessment by recognizing the developmental characteristics of professional evaluators assessing content for children aged 6–12 and creating design experiences that integrate educational content directly into age-appropriate interface mechanics. This comprehensive approach to Digital Game-Based Learning interface design establishes a framework for creating effective educational technology that acknowledges unique developmental interface needs while adapting to diverse educational contexts.

Future research must validate whether design engagement effectiveness translates into educational transformation and behavioral change through direct implementation studies with primary school students. The design validation approach provides critical groundwork for subsequent educational effectiveness research while establishing evidence-based interface optimization principles for primary school digital health education that could yield long-term public health benefits through effective early intervention design strategies.

## Figures and Tables

**Table 1:** Descriptive Statistics for Evaluator Demographics and Design Engagement Assessment (*N* = 337).

Variable	M	SD	Min	Max	Mdn	Skewness	Kurtosis
Age (years)	38.64	14.04	18	66	41	−0.27	−0.83
Steps completed	22.31	23.79	0	53	8	0.31	−1.85
Progress ratio	0.41	0.43	0	0.96	0.15	0.47	−1.74

Note. Progress ratio was calculated as steps completed divided by the total possible steps (55).

**Table 2:** Design Evaluator Distribution by Country.

Country	*N*	%	95% CI
Romania	209	62.0	[56.8, 67.2]
Cyprus	52	15.4	[11.5, 19.3]
Greece	38	11.3	[7.9, 14.7]
Spain	35	10.4	[7.1, 13.7]
Poland	3	0.9	[0, 1.9]

Note. CI = confidence interval.

**Table 3:** Bimodal Distribution of Design Exploration Patterns.

Progress Ratio	*n*	%	Cumulative %
0.0–0.2 (Limited exploration)	178	52.8	52.8
0.2–0.4 (Basic assessment)	27	8.0	60.8
0.4–0.6 (Moderate assessment)	3	0.9	61.7
0.6–0.8 (Extended assessment)	10	3.0	64.7
0.8–1.0 (Comprehensive assessment)	119	35.3	100.0

Note. Exploration ratio ranges represent different levels of design assessment depth conducted by teacher–evaluators.

**Table 4:** Age-Based Design Engagement Patterns with Cohen’s d Effect Sizes.

Age Group	*n*	Steps Completed			Progress Ratio		Cohen’s d
		M	SD	95% CI	M	SD	
18–25	55	21.45	22.87	[15.20, 27.70]	0.39	0.42	-
26–35	42	24.83	23.15	[17.61, 32.05]	0.45	0.42	0.14
36–50	183	22.55	24.16	[19.02, 26.08]	0.41	0.44	0.05
51–65	52	20.10	24.33	[13.32, 26.88]	0.37	0.44	−0.05

Note. Cohen’s d values represent effect sizes compared to the 18–25 age group. CI = confidence interval.

**Table 5:** Cross-National Comparison of Design Exploration Ratios with Post-Hoc Significance Testing.

Country	*n*	Progress Ratio			Significant Differences
		M	SD	95% CI	
Romania	209	0.460	0.44	[0.399, 0.521]	A
Poland	3	0.370	0.41	[0.000, 0.791]	a,b
Spain	35	0.337	0.42	[0.203, 0.471]	a,b
Greece	38	0.315	0.40	[0.190, 0.440]	b
Cyprus	52	0.301	0.38	[0.196, 0.406]	b

Note. Countries that do not share the same letter suffix differ significantly according to Tukey’s HSD test at *p* < 0.05. CI = confidence interval.

**Table 6:** One-Way ANOVA Results for Between-Country Differences in Design Exploration Ratios.

Source	*SS*	*df*	*MS*	*F*	*p*	*η* ^2^
Between groups	1.26	4	0.315	4.37	0.002	0.050
Within groups	23.94	332	0.072			
Total	25.20	336				

Note. *η^2^* = eta squared effect size.

**Table 7:** Pearson Correlations Between Key Design Assessment Variables with Bootstrapped 95% Confidence Intervals.

Variables	*R*	95% CI	*p*
Steps completed—Time spent	0.95	[0.93, 0.97]	<0.001
Age—Steps completed (full sample)	0.01	[−0.10, 0.12]	0.859
Age—Steps completed (adult subgroup)	0.80	[0.73, 0.85]	<0.001
Age—Time spent (adult subgroup)	0.60	[0.50, 0.69]	<0.001
Design exploration ratio—Time spent	0.94	[0.91, 0.96]	<0.001

Note. Confidence intervals were calculated using bootstrapping with 1000 samples. CI = confidence interval.

**Table 8:** Multiple Regression Analysis Predicting Design Exploration Ratio from Age, Country, and Initial Engagement.

Predictor	*B*	*SE B*	β	*t*	*p*	95% CI
Constant	0.210	0.074		2.84	0.005	[0.065, 0.355]
Age	0.003	0.002	0.108	1.97	0.049	[0.000, 0.006]
Initial engagement *	0.346	0.038	0.479	9.11	<0.001	[0.271, 0.421]
Country: Romania ^†^	0.159	0.048	0.183	3.31	0.001	[0.065, 0.253]
Country: Spain ^†^	0.036	0.065	0.026	0.55	0.580	[−0.092, 0.164]
Country: Greece ^†^	0.014	0.063	0.010	0.22	0.825	[−0.110, 0.138]
Country: Poland ^†^	0.069	0.165	0.019	0.42	0.676	[−0.256, 0.394]

Note. R^2^ = 0.31, F(6, 330) = 24.36, *p* < 0.001. CI = confidence interval. * Initial engagement measured by completion of the first 10 assessment steps (binary: yes/no) ^†^ The reference category is Cyprus.

**Table 9:** Cluster Analysis Results: Design Evaluator Typologies Based on Assessment Patterns.

Cluster	*n*	Age		Steps Completed		Progress Ratio		Time Spent (min)		Key Characteristics
		M	SD	M	SD	M	SD	M	SD	
Comprehensive design assessors	119	39.8	12.6	52.2	1.6	0.95	0.03	987.4	463.2	Complete most/all modules
Initial design explorers	178	38.1	14.9	4.5	3.8	0.08	0.07	65.3	52.8	Disengage after initial exploration
Selective design evaluators	40	37.9	13.5	29.3	7.1	0.53	0.13	384.6	159.7	Complete specific topics of interest

Note. Clusters were identified using the k-means clustering algorithm based on progress ratio and engagement pattern variables.

## Data Availability

The data presented in this study are available from the corresponding author upon request.
